# A community-based intervention (the Omama Project) improves neurodevelopment in impoverished 2-year-old Roma children: a quasi-experimental observational study

**DOI:** 10.1007/s00431-024-05967-9

**Published:** 2025-01-14

**Authors:** M. Fernandes, O. Matuskova, R. Babelova, W. B. Santosa, O. Shaw, P. Hrica

**Affiliations:** 1 Department of Paediatrics, Institute of Developmental and Regenerative Medicine, University of Oxford, Oxford, UK; 2 Oxford Maternal and Perinatal Health Institute, Green Templeton College, University of Oxford, Oxford, UK; 3 Nuffield Department of Women’s and Reproductive Health, John Radcliffe Hospitals, University of Oxford, Oxford, UK; 4 MRC Lifecourse Epidemiology Centre, University of Southampton, Southampton, UK; 5 National Institute of Children’s Diseases, Bratislava, Slovakia; 6 CESTA VON, Bratislava, Slovakia

**Keywords:** Roma, Early child development, Neurodevelopment, Intervention, Neurodevelopmental delay, INTER-NDA

## Abstract

High rates of childhood neurodisability are reported among the Roma, Europe’s largest ethnic minority community. Interventions targeting early child development (ECD) during the first 2 years of life can improve neurodevelopmental outcomes in vulnerable children; however, evidence from Roma preschoolers is scarce. In a quasi-experimental observational study, we compared neurodevelopmental outcomes at age 2 years, measured on the INTERGROWTH-21st Project Neurodevelopmental Assessment (INTER-NDA), between Roma children receiving a community-based ECD intervention (RI, *n* = 98), and age- and sex-matched Roma and non-Roma children (RC, *n* = 99 and NRC, *n* = 54, respectively) who did not receive the intervention in Eastern Slovakia. The intervention was delivered between 3 weeks and 20 months in weekly home-based sessions by trained Roma women from matched settlements to RIs. Compared with RC, RI had higher 2-year cognitive (*B* = 0.15; 95% CI, 0.04, 0.25), language (*B* = 0.25; 95% CI, 0.11, 0.38) and fine motor (*B* = 0.08; 95% CI, 0.01, 0.16) scores. After adjustment for covariates, cognitive delay decreased by 88% in RI compared with RC (aOR, 0.12; 95% CI, 0.03, 0.53). Linear growth at 24 months was a key predictor of developmental scores for both groups (range, *B* = 0.04–0.14; 95% CI, 0.01, 0.07 and 0.09, 0.20).

*Conclusions*: Our results highlight that, without directly intervening on nutritional and poverty status, a community-based ECD intervention, delivered by trained Roma women to Roma children, can significantly improve neurodevelopmental outcomes at age 2 years.

**Table:** 

**What is Known:** • *The Roma are Europe’s largest ethnic minority. High rates of neurodisability, malnutrition and poverty are reported in Roma preschoolers.* • *Optimal early child development (ECD) is foundational to lifecourse health and wellbeing. Early interventions improve ECD outcomes in vulnerable children; however, evidence from Roma communities is limited.*
**What is New:** • *The Omama project is a community-based ECD intervention, delivered by trained Roma women to Roma children aged 3 weeks to 20 months living in impoverished settlements in Eastern Slovakia.* • *Roma children receiving the intervention had (i) higher cognitive, language and fine motor scores and (ii) lower rates of cognitive delay compared with controls.*

**Supplementary Information:**

The online version contains supplementary material available at 10.1007/s00431-024-05967-9.

## Introduction

Optimal early child development (ECD) is foundational to childhood and adult health and wellbeing and is a necessary component of the Sustainable Development Goals. Poverty, stunting and early neurodevelopmental delays (ENDs) present a “triple threat” to at least 250 million children globally, preventing them from achieving their full developmental potential by 5 years [1]. Poverty is a key driver of both stunting and ENDs [2]. Recent evidence has shown that ENDs and their consequences on lifecourse health and wellbeing persist into subsequent generations [3, 4] resulting in vicious, perpetuating cycles of generational poverty and loss of developmental potential [5].

New research has shown that neurobiological, epigenetic and psychological adaptations to early environments can confer or mitigate END risk [6–9], with the first 1000 days of life—the period from conception to age 2 years—being of particular importance. Approaches targeting caregivers and children with effective interventions during this sensitive window of brain development have been shown to have positive and enduring results across the lifecourse [10, 11]. These form the basis of the United Nations’ 2018 Nurturing care framework [6].

While considerable ECD research has been undertaken in children from low- and middle-income countries, fewer inquiries focus on vulnerable populations of children living in poverty in high-income countries. The Roma community is Europe’s largest ethnic minority, estimated at 10–12 million [12, 13]. Roma families are often disadvantaged by lower education; social exclusion and discrimination, unemployment; financial struggles; poor health outcomes including high levels of substance abuse and mental health problems; and lack of access to basic household facilities [14–16]. Over 90% of European Roma children live in households that fall below the poverty line [17]. Compared with their non-Roma peers, Roma children under five are five times more likely to be malnourished [17–20]; 60% more likely to have mild to moderate neurodisability; and twice as likely to have a severe disability following risk adjustments for age and gender [21, 22]. Roma children therefore experience the “triple threat” of poverty, stunting and ENDs, and many do not achieve their full developmental potential by age five. In addition, many Roma children face discrimination and exclusion on the basis of their ethnicity [23].

While some European and national strategies target preschool education, second language learning and social inclusion in Roma children, there are, as yet, no interventions specifically targeting ECD in this group during the critical “first 1000 days of life” window [24, 25]. The Omama Project, led by non-governmental organisation CESTA VON based in Slovakia, is a community-based social and educational package that provides young Roma children with a holistic ECD intervention delivered by trained Roma women from matched settlements.

Our objective was to determine whether ECD outcomes at age 2 years differed between Roma children who received the Omama intervention (Roma intervention group, RI), and age- and sex-matched Roma and Non-Roma children (Roma controls, RCs and Non-Roma controls, NRCs, respectively) who did not receive the intervention. The study’s aims were to (i) compare neurodevelopment scores and rates of ENDs at age 2 years between the three groups and (ii) to examine whether these differences, if any, persisted between RI and RC groups after adjusting for baseline differences in early life exposures (ELEs) and growth indices.

## Methods

### Study design and procedure

The Omama Project is a multi-site, ECD and social intervention project targeted to Roma children living in poverty in marginalised settlements in Slovakia (https://cestavon.sk/en/omama/). RIs were enrolled into the Omama intervention between 2019 and 2021. They received the intervention once a week until age 2 years. Neurodevelopment and growth outcomes were assessed at 2 years using standardised protocols. Age- and sex-matched RC and NRC were also assessed at 2 years using identical ECD and growth measures.

A non-randomised, cross-sectional, observational design was used to compare (i) neurodevelopmental scores, (ii) rates of severe and mild-to-moderate ENDs at age 2 years, between children who received the intervention (RI) and who did not (RC and NRC). This design was selected because the Omama intervention was already implemented by CESTA VON as a community-based social and educational intervention project before the 2-year ECD outcome assessment was designed [26]. Moreover, it was not possible to randomise children between groups for all key factors known to be associated with ENDs. Rather, we collected information on key ELEs and growth metrics known to be associated with ENDs and adjusted for those that differed significantly between RC and RI in our analyses.

### Setting

Study sites (Supplementary Information Figure S1) included eleven Roma settlements from the Banská Bystrica, Prešov and Košice regions in Eastern and Central Slovakia. NRCs were recruited from eight cities in these regions.

### Participants and eligibility

RI and RC were identified from participating settlements by Omamas at between 3 weeks and 20 months and 22–26 months, respectively. They were eligible to participate if they were fluent in either the local Romani dialect or Slovak. Children with siblings receiving the intervention were excluded. NRC were identified from local preschools in the same regions and included if they were aged 22 to 26 months and fluent in Slovak. In all groups, children with known life limiting conditions were excluded.

### The Omama intervention

The Omama intervention is a holistic multi-modal ECD intervention which includes age-specific ECD stimulation activities delivered by trained Roma women (termed “Omamas”, i.e. Slovak for “grandmothers”) to Roma children aged 3 weeks to 24 months. It is delivered in weekly, 1-h, sessions in the child’s home in the presence of the primary caregiver who is trained in the activities and encouraged to continue these during the week. Omamas’ obtain a pre-defined schedule of age-appropriate activities via the Project’s mobile-device-based application: these include aspects of kangaroo mother care, infant massage, play, reading, music and responsive caregiving (Supplementary Information Table S2). Activities are conducted in the local Romani dialect. The intervention also includes teaching the children Slovak as a second language.

Omamas are recruited from participating settlements and trained to deliver the intervention using a participatory, peer-to-peer approach. Further details on their training, ongoing mentorship and supervision and adherence to protocol are presented in Supplementary Information S3. As Romani dialects may differ between settlements, Omamas deliver the intervention to children residing in the same settlement as themselves.

### Measures

ECD and growth assessments were undertaken at 2 years using the INTERGROWTH-21st Project and WHO protocols, respectively. Demographic and ELE information including perinatal, health, socio-economic and family factors (namely, preterm birth (defined as birth before 37 completed gestational weeks), pregnancy-related complications, maternal and paternal education and employment and housing conditions) were collected using caregiver interviews.

All assessors were trained and standardised centrally in the ECD and growth assessments. Assessors included both Omamas and local specialists (psychologists and paediatricians). Omamas did not assess children from their settlements and to whom they had provided the intervention. All assessors were blind to the intervention status of the children.

I. **The INTERGROWTH-21st Neurodevelopmental Assessment (INTER-NDA): **The INTER-NDA (www.inter-nda.com) is a standardised, psychometrically valid, international, rapid ECD assessment for children aged 22 to 30 months [18, 23]. Its 37 items measure cognitive, language, fine and gross motor and positive and negative behaviour outcomes utilising a mixed methodology approach including direct testing, concurrent observation and caregiver reports. It is administered reliably by non-specialist assessors in an assessment time of 15–20 min [18]. Its norms are international ECD standards, rather than population-specific references, constructed from five low-risk, international populations according to the WHO MGRS’ prescriptive guidelines [18]. For all domains, except negative behaviour, higher INTER-NDA scores reflect better outcomes and no, any, mild-to-moderate and severe delay are defined as > 10th, < 10th, 3rd–10th and < 3rd centiles, respectively [18]. For negative behaviour, lower scores reflect better outcomes and no, any, mild-to-moderate and severe problems are defined as < 90th, > 90th, 90th–97th and > 97th centiles, respectively [18].
II. **Growth and Nutritional Assessment:** Anthropometric measurements (weight, length and head circumference) were undertaken according to the WHO MGRS protocol and converted to age- and sex-adjusted *z* scores based on the WHO standards [22].

### Sample size estimations and power calculations

Based on a prevalence estimate of severe END of 20%, and a medium effect size of 0.5, a sample size of 82 children in the intervention and control groups was estimated. This provides the study with power above 90% at the significance level of 5%. Accounting for attrition (between intervention commencement and outcome assessment) at 20%, a final sample size of 98 RI children was estimated. Attrition rates were not applicable to control groups.

### Statistical analysis

Statistical analysis was performed in IBM SPSS V.29.0. For all ECD outcomes, primary comparisons were undertaken between RI and RC and secondary comparisons were made between RI, RC and NRC.

Summary statistics for ELEs and growth outcomes were compared using chi-square tests, independent sample *t*-tests and one-way analysis of variance (ANOVAs) as appropriate. Age- and sex-adjusted *z* scores for weight, length, BMI and weight for length were calculated using the WHO Anthro Survey Analyser [27] and compared between groups.

INTER-NDA raw domain scores were converted into standardised domain scores and compared with the INTER-NDA international standards [28].

The distributions of ECD outcomes were inspected visually and confirmed using the Kolmogorov–Smirnov test and Q-Q plots. These were not normally distributed, and no transformation was identified that suited all ECD outcomes; therefore, we used non-parametric tests to undertake unadjusted primary and secondary comparisons for INTER-NDA standardised domain scores. END rates, categorised as (i) no delay vs any delay and (ii) no delay, mild to moderate delay and severe delay, were compared between the groups using chi-square tests.

We used regression analyses to determine whether the association between group assignment (RI vs RC) and ECD outcomes persisted after adjusting for baseline differences in ELE and growth covariates. The use of a generalised linear model approach with a gamma log link function (due to the positive skew of INTER-NDA scores) allowed the assessment of differences in INTER-NDA standardised domain scores between groups RC and RI, while adjusting for pertinent covariates (namely, group, age at INTER-NDA assessment and mode of delivery). Ordinal outcomes (i.e. severe, mild to moderate and no delay) were analysed using logistic regression with adjustments made for the same covariates. For all analyses, we considered a probability (*p*) value < 0.05 as significant.

## Results

### Characteristics of the study population

Two hundred and fifty-one children completed the 2-year assessment. The proportionate group contribution to the sample was RC *n* = 99 (39.44%), RI *n* = 98 (39.04%) and NRC *n* = 54 (21.51%) (Table 1). The age at INTER-NDA assessment was 25.34 months (SD 2.10). Just over half the sample (*n* = 128, 50.99%) were boys; across groups, INTER-NDA standardised scores in all six domains did not differ between boys and girls (*U* = 7019.5–7892.0, *p* = 0.06–0.97).

**Table 1:** Pre-, peri- and postnatal health and growth characteristics of children in the Omama project cross-sectional ECD study

Pre-, peri- and postnatal health and growth characteristics	Pooled sample (*n* = 251)	Roma control (RC) group (*n* = 99)	Roma intervention (RI) group (*n* = 98)	Non-Roma control (NRC) group (*n* = 54)	Test statistic, *p* value	
					RC vs RI comparison	RC, RI v NRC comparison
Socio-demographic characteristics (mean (SD) or number (%))						
Sex, males (*n* (%))	128 (50.9%)	46 (46.5%)	50 (51.0%)	32 (59.3%)	*X*^2^ = 0.4, *p* = 0.52	*X*^2^ = 2.3, *p* = 0.32
Age at INTER-NDA assessment (mean (SD))	25.3 (2.1)	24.9 (2.7)	25.8 (1.7)	25.3 (1.4)	*t* = − 2.9, *p* = 0.02*	*F* = 4.9, *p* = 0.007*
Age at enrollment into the Omama Intervention (in months, mean (SD))	NA	NA	10.0 (7.1)	NA	NA	NA
Total number of Omama intervention sessions (mean (SD))	NA	NA	27.2 (15.9)	NA	NA	NA
Pre- and perinatal characteristics (mean (SD) or number (%))						
Complications during pregnancy (*n* (%))	21 (8.4%)	6 (6.1%)	8 (8.3%)	7 (13.2%)	*X*^2^ = 2.3, *p* = 0.32	*X*^2^ = 5.2, *p* = 0.27
Mode of delivery (*n* (%)):						
NVD:	215 (86.0%)	90 (90.9%)	78 (78.6%)	47 (8.7%)	*X*^2^ = 9.2, *p* = 0.03*	*X*^2^ = 11.0, *p* = 0.09
C-section:	35 (14.0%)	8 (8.1%)	20 (20.4%)	7 (1.3%)		
Missing *n* = 1						
Preterm birth < 37 gestational weeks	7 (2.8%)	3 (3.1%)	4 (4.1%)	0 (0%)	*X*^2^ = 0.2, *p* = 0.70	*X*^2^ = 2.2, *p* = 0.34
Postnatal health and growth characteristics at 24 months (mean (SD) or number (%))						
Maternally report child health problems during the first 2 years (*n* (%))	11 (4.4%)	5 (5.2%)	6 (6.2%)	0 (0%)	*X*^2^ = 0.1, *p* = 0.76	*X*^2^ = 3.3, *p* = 0.19
Head circumference (in cm, mean (SD))	47.2 (1.8)	46.6 (1.5)	46.9 (1.5)	48.8 (1.5)	*t* = − 1.2, *p* = 0.11	*F* = 39.1, *p* < 0.001*
Weight (in kg, mean (SD))	11.6 (1.6)	11.4 (1.5)	11.3 (1.7)	12.1 (1.4)	*t* = 0.5, *p* = 0.31	*F* = 11.7, *p* < 0.001*
Length (in cm, mean (SD))	83.2 (4.8)	81.0 (4.3)	83.1 (4.3)	87.5 (3.9)	*t* = 3.3, *p* < 0.001*	*F* = 41.0, *p* < 0.001*
Weight *z* score (mean (SD))	− 0.4 (1.2)	− 0.4 (0.1)	− 0.7 (0.1)	0.3 (0.9)	*t* = 1.5, *p* = 0.06	*F* = 12.2, *p* < 0.001*
Length *z* score (mean (SD))	− 1.3 (1.4)	− 1.9 (1.4)	− 1.5 (1.2)	0.01 (1.2)	*t* = − 2.4, *p* = 0.008*	*F* = 42.2, *p* < 0.001*
Weight for length *z* score (mean (SD))	0.4 (1.2)	0.8 (1.1)	0.1 (1.3)	0.3 (1.1)	*t* = 3.6 *p* < 0.001*	*F* = 7.2, *p* < 0.001*
BMI *z* score (mean (SD))	0.7 (1.3)	1.1 (1.2)	0.4 (1.4)	0.3 (1.2)	*t* = 4.1, *p* < 0.001*	*F* = 10.8, *p* < 0.001*

^*^p < 0.05

*NA* not applicable for RC and RI groups

For RI, children were enrolled at 10.03 months (SD 7.09) and received a mean of 27.15 (SD 15.94) intervention sessions. Age at enrolment and number of intervention sessions were significantly correlated (*r* = − 0.72, *p* < 0.001).

Compared with RCs, RIs were older with greater lengths at the INTER-NDA assessment and had increased rates of births by C-section (Table 1). Length-associated growth metrics including length z score, weight for length *z* score and BMI z score differed significantly between the groups (Table 1). NRC showed the best and RC showed the least overlap with the WHO child growth standards (Supplementary Information Figure S3). No differences in pregnancy-related complications, preterm birth, maternally reported health problems in the children and weight at 2 years were observed. NRC differed significantly from RC and RI across all growth indices. Family and housing indicators were very similar between RC and RI groups but differed significantly, across most indicators, between Roma children and NRC (Supplementary Information Table S5).

All children enrolled in the study completed the 2-year neurodevelopmental assessment. For RC and NRC, this was a cross-sectional, one-off assessment, and therefore, attrition was not relevant. All enrolled RI children received the Omama intervention consistently until the point of the 2-year neurodevelopmental assessment; none dropped out from the program.

### Comparisons in ECD outcomes between study groups

#### *INTER-NDA scores*

**Fig. 1:**
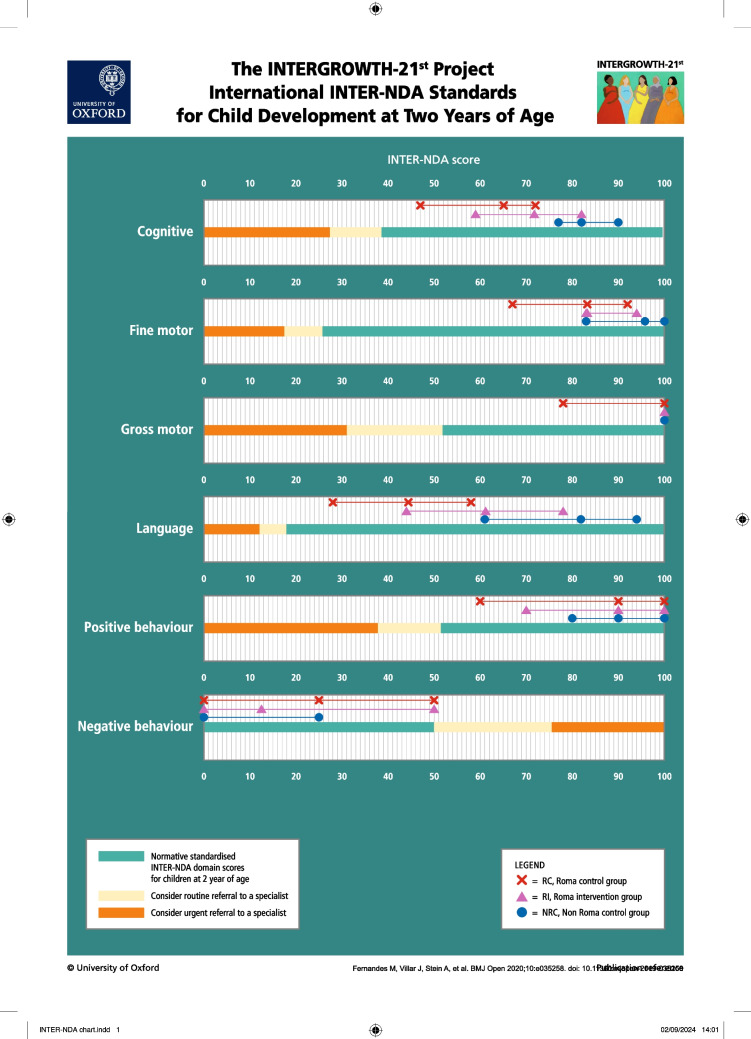
Comparison of INTER-NDA domain scores between groups according to the INTER-NDA international standards

In unadjusted primary comparisons, RI had significantly higher scores for cognitive, language, fine motor and gross motor domains than RC (Table 2). In secondary comparisons, RI had significantly lower cognitive, language, fine motor and gross motor scores than NRC, while RC had significantly lower scores than NRC across all domains except negative behaviour (Table 2, Fig. 1 and Supplementary Information Table S6).

**Table 2:** Unadjusted comparisons: Neurodevelopmental scores of children in the Omama Project cross-sectional ECD study at age 2 years

INTER-NDA standardised domain scores (median, IQR)	Pooled sample	Roma control (RC) group (*n* = 99)	Roma intervention (RI) group (*n* = 98)	Non-Roma control (NRC) group (*n* = 54)	Test statistic, *p* value		
					RC vs RI comparison	NRC vs RI comparison	RC, RI v NRC comparison
Cognition^1^	71.8 (23.1)	64.1 (24.6)	71.8 (23.4)	82.1 (12.8)	*U* = 6673.0, *p* < 0.001*	*U* = 3860.0, *p* < 0.001*	*F* = 41.3, *p* < 0.001*
Language^1^	57.6 (39.4)	44.4 (30.6)	61.1 (33.6)	81.94 (33.5)	*U* = 6753.5, *p* < 0.001*	*U* = 3785.5, *p* < 0.001*	*F* = 39.7, *p* < 0.001*
Fine motor^1^	83.3 (13.9)	83.3 (25.0)	83.3 (10.4)	95.83 (16.7)	*U* = 5988.5, *p* = 0.004*	*U* = 3446.5, *p* = 0.001*	*F* = 18.6, *p* < 0.001*
Gross motor^1^	100.0 (11.1)	100.0 (22.2)	100.0 (0.0)	100.0 (0.0)	*U* = 6078.0, *p* < 0.001*	*U* = 2939.5, *p* = 0.10	*F* = 14.4, *p* < 0.001*
Positive behaviour^1^	90.0 (30.0)	90.0 (40.0)	90.0 (30.0)	90.0 (20.0)	*U* = 5115.0, *p* = 0.484	*U* = 2792.5, *p* = 0.55	*F* = 3.1, *p* = 0.04*
Negative behaviour^2^	0.0 (25.0)	25.0 (50.0)	12.5 (50.0)	0.0 (25.0)	*U* = 4658.0. *p* = 0.60	*U* = 2284.5, *p* = 0.13	*F* = 2.7, *p* = 0.07

^1^For these INTER-NDA domains, higher scores reflect better outcomes

^2^For negative behaviour, lower scores reflect better outcomes

The association between the intervention group and higher INTER-NDA cognitive, language and fine motor scores persisted after adjusting for baseline differences in covariates between RC and RI (Table 3). Length *z* score remained a significant predictor for these domains, as well as for gross motor and positive behaviour.

**Table 3:** Generalised linear regression models for INTER-NDA scores after adjusting for significant covariates

INTER-NDA domain	Parameter	Parameter estimates				Hypothesis test		
		*B*	Std. error	95% Wald confidence interval				
				Lower	Upper	Wald chi-square	Df	Sig
Cognition	Omama intervention (RI vs RC)	0.15	0.05	0.04	0.25	7.78	1	0.005*
	Mode of delivery (C-section)	− 0.08	0.08	− 0.23	0.07	1.16	1	0.28
	Length *z* score	0.10	0.02	0.06	0.14	20.52	1	< .001*
	Age at assessment in months	0.02	0.01	0.00	0.04	3.90	1	0.04*
Language	Omama intervention (RI vs RC)	0.25	0.07	0.11	0.38	12.90	1	< .001*
	Mode of delivery (C-section)	0.05	0.10	− 0.15	0.24	0.21	1	0.645
	Length *z* score	0.14	0.03	0.09	0.20	27.15	1	< .001*
	Age at assessment in months	0.04	0.01	0.02	0.07	8.92	1	0.003*
Fine motor	Omama intervention (RI vs RC)	0.08	0.04	0.01	0.16	5.24	1	0.02*
	Mode of delivery (C-section)	0.03	0.05	− 0.08	0.13	0.28	1	0.60
	Length *z* score	0.05	0.01	0.02	0.08	12.78	1	< .001*
	Age at assessment in months	0.01	0.01	− 0.003	0.03	2.35	1	0.13
Gross motor	Omama intervention (RI vs RC)	0.08	0.04	− 0.001	0.16	3.75	1	0.05
	Mode of delivery (C-section)	− 0.03	0.06	− 0.14	0.09	0.17	1	0.68
	Length *z* score	0.04	0.02	0.01	0.07	6.04	1	0.01*
	Age at assessment in months	0.01	0.01	− 0.004	0.03	2.14	1	0.14
Positive behaviour	Omama intervention (RI vs RC)	0.02	0.06	− 0.10	0.14	0.09	1	0.75
	Mode of delivery (C-section)	− 0.01	0.09	− 0.18	0.15	0.03	1	0.87
	Length *z* score	0.05	0.02	0.002	0.10	4.17	1	0.04*
	Age at assessment in months	0.00	0.01	− 0.03	0.03	0.00	1	0.98
Negative behaviour	Omama intervention (RI vs RC)	0.003	0.10	− 0.20	0.21	0.001	1	0.98
	Mode of delivery (C-section)	0.15	0.13	− 0.10	0.41	1.39	1	0.24
	Length *z* score	− 0.004	0.03	− 0.07	0.06	0.02	1	0.89
	Age at assessment in months	− 0.03	0.03	− 0.08	0.02	1.35	1	0.24

^*^p < 0.05

*RI* Roma intervention group, *RC* Roma control group

#### *Neurodevelopmental delay*

Rates of *any* cognitive, language and gross motor delay were significantly lower in RI than in RC (Supplementary Information Table S7). Across all domains, rates of *any* delay did not differ significantly between RI and NRC.

**Fig. 2:**
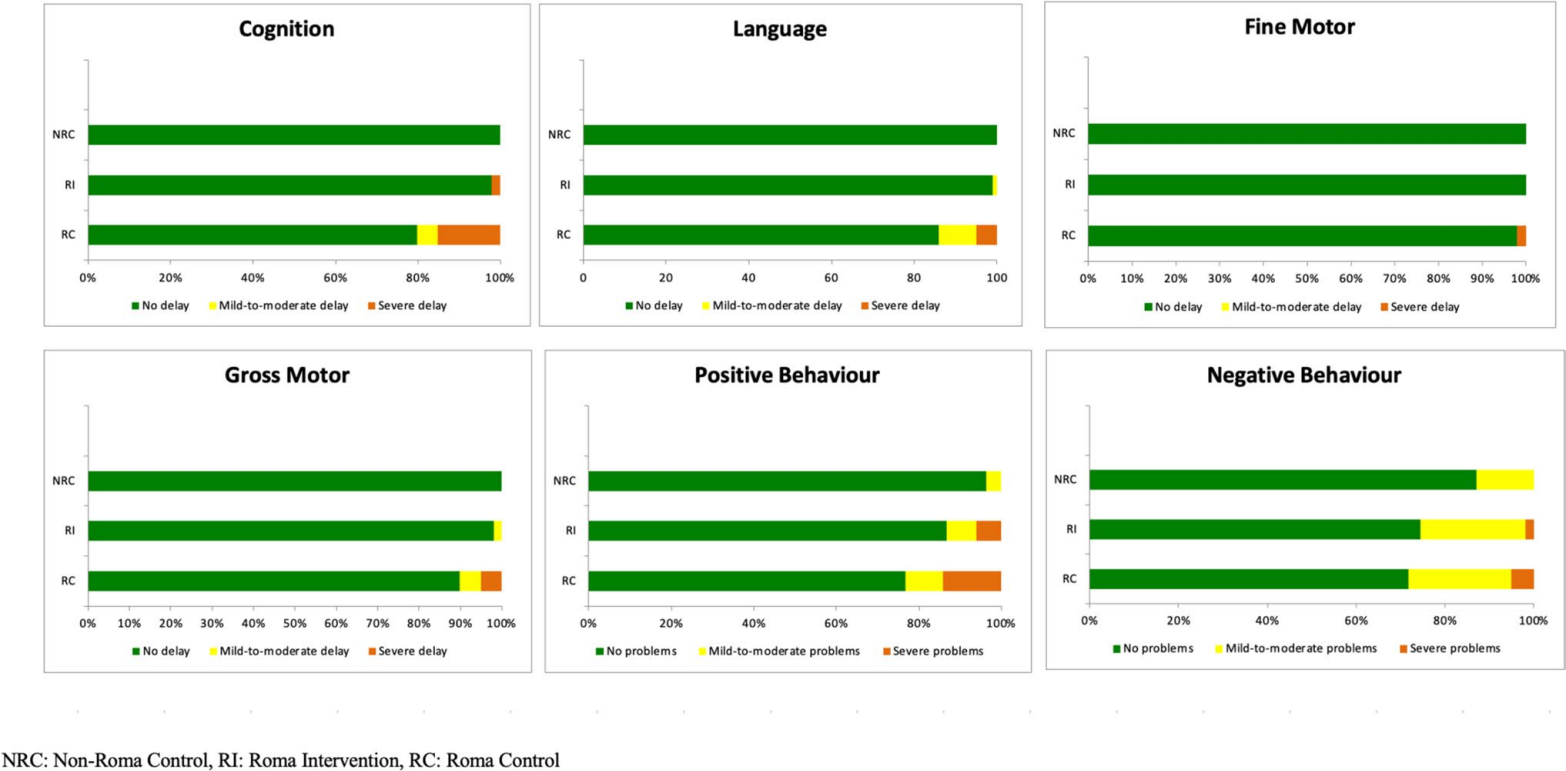
Comparison of rates of delay by domain and severity between groups according to the INTER-NDA international standards

The distribution of severe, mild to moderate and no delay for each group and domain is presented in Fig. 2 and Supplementary Information Table S8. Compared with RC, RI showed significantly lower rates of severe and mild-to-moderate delays for cognitive, language and gross motor domains. Although a similar pattern was observed for positive and negative behaviour problems, the differences were not statistically significant. The association between the intervention group and cognitive delay persisted after adjusting for baseline differences in covariates between the groups (Table 4). Rates of mild-to-moderate and severe delay across domains did not differ significantly between RI and NRC (Supplementary Information Table S8).

**Table 4:** Ordinal logistic models for rates and severity of delays between Roma intervention and control groups after adjusting for significant covariates

INTER-NDA domain	Parameter	OR	95% CI		Sig	aOR	95% CI		Sig
			Lower	Upper			Lower	Upper	
Cognition	Omama intervention (RI vs RC)	0.08	0.02	0.37	0.001*	0.12	0.03	0.53	0.006*
	Mode of delivery (C-section)	0.27	0.04	2.09	0.21	0.21	0.02	2.49	0.21
	Length *z* score	0.38	0.25	0.58	< 0.001*	0.42	0.27	0.66	< 0.001*
	Age at assessment in months	0.77	0.58	1.01	0.06	0.89	0.73	1.08	0.25
Language	Omama intervention (RI vs RC)	0.06	0.01	0.48	0.008*	NA	NA	NA	NA
	Mode of delivery (C-section)	0.43	0.06	3.40	0.43	0.53	0.05	5.56	0.60
	Length *z* score	0.38	0.24	0.61	< 0.001*	0.45	0.26	0.76	0.003*
	Age at assessment in months	0.90	0.67	1.20	0.48	0.96	0.78	1.17	0.66
Gross motor	Omama intervention (RI vs RC)	0.18	0.04	0.85	0.03*	0.26	0.05	1.31	0.10
	Mode of delivery (C-section)	1.25	0.26	5.96	0.78	1.63	0.29	9.26	0.58
	Length *z* score	0.40	0.25	0.65	< 0.001*	0.46	0.27	0.79	0.004*
	Age at assessment in months	0.74	0.51	1.08	0.12	0.85	0.62	1.16	0.31

^*^p < 0.05

*NA* not applicable, *OR* odds ratio, *aOR* adjusted odds ratio, *RI* Roma intervention group, *RC* Roma control group

## Discussion

Our results show that (i) a community-based ECD intervention, initiated during the first two years of life, and delivered by trained Roma women to Roma children living in impoverished settlements in Eastern Slovakia, is associated with improved ECD outcomes and reduced rates of delay at 2 years and that (ii) despite improvements in ECD, RI did not achieve the same outcomes as NRCs suggesting that additional interventions, such as those addressing malnutrition, may be needed. We found that, compared with RC, RI had significantly better INTER-NDA scores across cognitive, language, fine motor and gross motor domains after adjusting for covariates. However, despite this difference, INTER-NDA scores were lower in RI than in NRC. We also found that, compared with RC, RI had significantly lower rates of severe, mild to moderate and any delays in cognitive, language and gross motor domains, however after adjustment for covariates only the association with cognitive delay remained significant. Linear growth at 24 months remained a key predictor of ECD outcomes after adjusting for covariates.

Our findings are comparable to those reported from community-based ECD interventions applied in low- and middle-income countries [2, 29–32] and in high-risk populations in the UK and USA [33, 34]. Improvements in cognitive, motor and language scores have been reported from community-based ECD and parenting intervention studies in low-resource settings [2, 32, 35, 36]. Similarly, we found higher cognitive, language and motor scores, and lower rates of delays in these domains, in RI compared with RC, while we did not detect differences in END rates between RI and NRC despite cognitive, language and motor scores being higher in NRC. A possible explanation for our results is that while our intervention was able to mitigate END risk and improve ECD scores to a significant extent in RI, the apparent catch-up was partial perhaps due to the co-occurring influence of other key drivers of early developmental delay present in this population and unaddressed by our intervention such as undernutrition. Stunting and micro-nutrient deficiencies, including iodine and iron deficiency, have been associated with ENDs and, when untreated, have been reported to attenuate the effect of parent–child-directed ECD interventions [37–39]. Moreover, domain-specific differences between groups were more apparent in directly assessed domains (cognition, language and motor skills) than in observer-rated domains (positive and negative behaviour).

However, despite their small to moderate effect sizes, community-based ECD interventions have been argued to be of policy significance as they are applied on a population-wide basis, have long-term downstream effects [10, 40] and are relevant to narrowing the gap in ECD and later neurocognitive, social and mental health outcomes between disadvantaged children and the rest of the population [33]. This is particularly important in the context of the Eastern Slovakia’s Roma community who experience high rates of poverty, malnutrition and neurodisability [20, 41]. Importantly, the most potent predictor of ECD in our sample was linear growth at 24 months highlighting the potential of malnutrition as a key driver of neurodisability in Roma children. Linear growth from early infancy into the second year of life has previously been associated with ECD outcomes, in both observational [42, 43] and interventional studies [44, 45].

Our results add significantly to the existing yet sparse evidence base on strategies to improve ECD in Roma children. They are one of the few studies published to date that (i) report on potential beneficial effects of a community-based ECD intervention, developed for and delivered by the Roma to their children within the first two years and (ii) compare ECD scores and rates of ENDs between Roma and non-Roma children on an international ECD standard [28]. Moreover, to our knowledge, this is the only study comparing ECD outcomes between RI and a positive (NRC) and negative (RC) control group. Importantly, the intervention was delivered by Roma women to children from their communities and therefore avoided some of the key methodological shortcomings that characterise much of the existing literature, including language and cultural aspects, and suspicion on the part of Roma parents about non-Roma personnel visiting their homes and interacting with their children [46]. The intervention was unique in that it was administered in children’s homes and employed a mixed-methodology approach incorporating aspects of kangaroo mother care (where appropriate), infant massage, play-based neurodevelopmental stimulation, reading, music and responsive caregiving.

Our results need to be considered in the context of several limitations. First, the study employed a quasi-experimental, cross-sectional observational design without randomisation of groups and without baseline ECD assessments at enrolment. A randomised controlled trial would have been the strongest evaluation strategy; however, (i) the Omama Project began and continues to be a social and educational intervention programme, rather than a research study, and (ii) it was not possible to randomise children between groups for all key factors known to be associated with ENDs. RI enrolment prior to the study design precluded the possibility of baseline assessments and randomisation. The design we adopted was the most robust possible given these constraints and has been previously employed for the evaluation of community-based interventions in high-income countries [33]. Information on maternal education, maternal mental health status and family household income was not collected as these were considered highly sensitive topics to Roma communities. While we achieved our sample size for RC and RI, we were unable to achieve this for NRC due to non-Roma parents being unwilling to consent to their children’s participation. We were unable to discern the reason for this. We were also unable to measure parental compliance with carrying out the session’s activities themselves with their children. Finally, while our results make it unlikely, we cannot rule out the possibility that RI mothers shared information about the intervention with RC mothers from their communities.

Roma children face multiple hardships as minorities in Europe, including high levels of racial discrimination and social exclusion [23], and have limited access to opportunities, during their early years, to promote and/or rescue their development. Here, we show that a community-based, culturally sensitive intervention, delivered through trained Roma women, has beneficial effects on neurocognitive outcomes in Roma children. Our findings have the potential to impact policy and practice, specifically in supporting the case for resource allocation, workforce development, investment into holistic early interventions and the development of integrated cross-sectoral policies to reduce inequities in children from marginalised communities. Future research is needed to (1) determine whether these ECD differences between groups persist into school-age and adolescent interventions and (2) evaluate the effect of a combined ECD and nutritional intervention using a cluster, randomised controlled design. This is critical to provide public health agencies with the necessary evidence and resources to embed these strategies in existing health and social programmes for the Roma and to promote population health in Europe.

## Conclusions

In this quasi-experimental, observational study, we have shown that a community-based, multi-modal and culturally sensitive intervention, delivered during the first 2 years by trained Roma women, has beneficial effects on improving neurocognitive outcomes and reducing rates of delay in 2-year-old Roma children from impoverished settlements in Eastern Slovakia. Linear growth at 24 months was an additional potent predictor of ECD outcomes. Further research incorporating randomised controlled designs and nutritional interventions is needed to develop a holistic, low-cost, community-led ECD intervention package for Roma children.

## Supplementary Information

Below is the link to the electronic supplementary material.

Supplementary file1 (DOCX 1397 KB)

## Data Availability

No datasets were generated or analysed during the current study.

## Abbreviations

BMI: Body mass index
ECD: Early child development
ELEs: Early life exposures
ENDs: Early neurodevelopmental delays
INTER-NDA: INTERGROWTH-21st Project Neurodevelopment Assessment
MGRS: Multi-centre Growth Reference Study
NRC: Non-Roma control group
RC: Roma control group
RI: Roma intervention group
