# Supplementary material for: A community-based intervention (the Omama Project) improves neurodevelopment in impoverished 2-year-old Roma children: a quasi-experimental observational study
Source: Eur J Pediatr. 2025 Jan 14;184(2):133. doi: 10.1007/s00431-024-05967-9 (PMC11732948; doi:10.1007/s00431-024-05967-9)
Supplement: Supplementary file 1 — Supplementary file1 (DOCX 1397 KB) [file 431_2024_5967_MOESM1_ESM.docx]

**List of Supplementary Information**

**Supplementary Information Figure S1** Map showing participating locations

**Supplementary Information Table S2** Early child development components of the Omama intervention

**Supplementary Information S3** Approach to training and mentoring of Omamas

**Supplementary Information Figure S4** Comparison of growth indices between groups according to the WHO International Child Growth Standards

**Supplementary Information Table S5** Family and home environment characteristics of children in the Omama project cross-sectional ECD study at age 2 years

**Supplementary Information Table S6** Three-group comparisons of neurodevelopmental scores of children in the Omama project cross-sectional ECD study at age 2 years

**Supplementary Information Table S7** Unadjusted comparisons**:** Developmental delay rates by domain at age 2 years

**Supplementary Information Table S8** Unadjusted comparisons**:** Developmental delay rates by domain and severity at age 2 years

**Supplementary Information Figure S1** Map showing participating locations

**
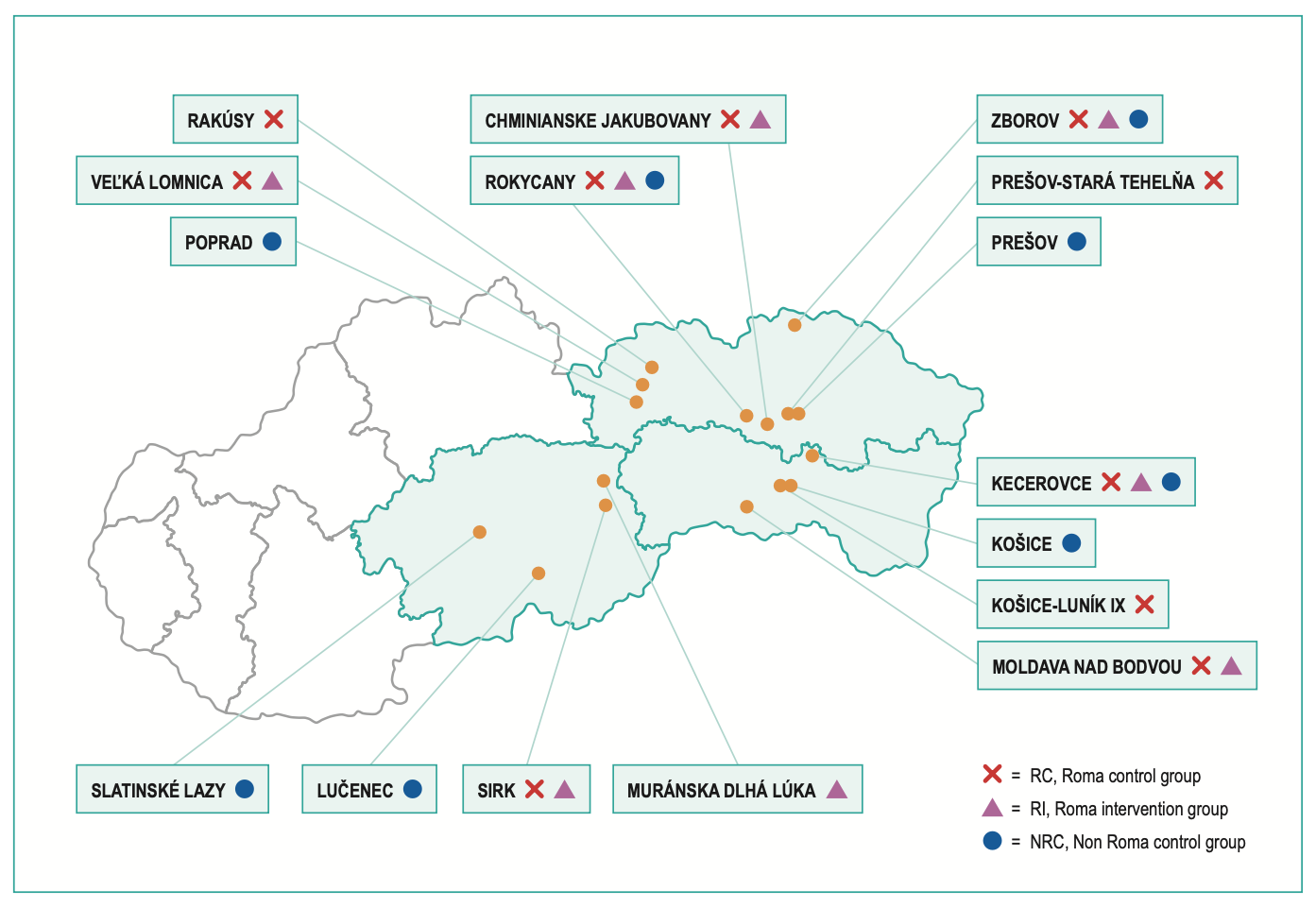
**

**Supplementary Information Table S2** Early child development components of the Omama intervention

| **Intervention component** | **Age range** | **Example activities** |
| --- | --- | --- |
| Kangaroo mother care | 0-12 weeks | Supporting mothers with providing skin to skin contact with their babies during the first three months Reference protocol: World Health Organization. Reproductive Health. Kangaroo mother care: a practical guide. World Health Organization; 2003 Apr 17. |
| Infant massage | 0-6 months | Teaching mothers techniques of infant massage and nurturing touch, including upper limbs, lower limbs, chest and abdomen, back and face massage. Reference protocol: International Association of Infant Massage |
| Play-based stimulation | 0-30 months | Teaching mothers play-based simulation activities, using principles of ‘serve and return’ and modelling play and stimulation of typical competencies of infants and toddlers.  Gross motor: tummy time, encouragement to crawl, supported steps, crawling through a tunnel, climbing stairs, kicking a ball, jumping on coloured tiles, running  Fine motor: holding objects in palm, picking up objects using pincer grasps, releasing pegs into holes, turning pages of a book, scribbling, threading beads  Cognition: following simple (one-, two- and three-stage) commands, matching and sorting according to shape, colour, size, number and object type, stacking, puzzle-based activities  Language: modelling and supporting the development of a range of verbal and non-verbal communication skills, naming colours, numbers, and objects in the local Romani dialect and Slovak, communicating needs  Personal Social: Lessons are structured to transform routine play into enhanced early experiences. Through engagement and modelling, Omamas demonstrate socially acceptable behaviour. Each activity takes the form of reciprocal social interaction that brings pleasure and simultaneously facilitates the regulation of behaviour. Omamas learn to actively listen to children, provide positive reinforcement, teach children to follow rules, to wait their turn and complete an activity before moving onto another. They encourage children and parents to communicate with each other and provide feedback during activities.  Problem solving: matching and sorting according to shape, colour, size, number and object type |
| Reading | 0-30 months | Supporting and encouraging mothers to read a simple story at least once a day to their child in either the local Romani district (if printed material is available) or in Slovak. |
| Music | 0-30 months | Supporting and encouraging mothers to sing to and with their children one or more times a day in either the local Romani district or in Slovak. Mothers are encouraged to use opportunities during her daily chores and activities during which to sing to her child, such as during cooking or whilst tidying the house. |
| Responsive Caregiving and positive parent-child interaction | 0-30 months | Supporting the development of mutually pleasant and rewarding interactions between parents and children, teaching mothers techniques of infant soothing, supporting mothers in understanding children’s cues, teaching mothers to respond to children in a sensitive way, teaching mothers skills by which they can encourage their child’s exploration and discovery activities in a positive manner, teaching mothers techniques of conscious parenting. |
| Promoting parental engagement |  | Promoting parental engagement through monthly parental meetings, education of parents regarding developmental milestones and opportunities for early learning, play-based developmental stimulation home assignments for parents. Building confidence in parenting skills through positive feedback and encouragement. |

Reference: Omama Manual, Bratislava © Cesta Von 2021

**Supplementary Information S3** Approach to training and mentoring of Omamas

The Omama intervention is a holistic multi-modal ECD intervention which includes age-specific ECD stimulation activities delivered by trained Roma women (termed “Omamas”, i.e. Slovak for “grandmothers”) to Roma children aged three weeks to 24 months. Omamas are recruited from participating settlements and trained to deliver the intervention using a participatory, peer-to-peer approach. As Romani dialects may differ between settlements, Omamas deliver the intervention to children residing in the same settlement as themselves.

**Recruitment of Participating Omamas:**

Omamas are carefully selected among well-established Roma women, most of whom are mothers themselves, from the same participating settlements as the children. They are familiar with the local context and community, and therefore have the trust and respect of their peers. They are employed either full or part-time to deliver the intervention by the NGO CESTA VON and they receive a salary for their efforts.

**Training Protocol:** As Omamas typically have very few years of formal education (usually having only completed elementary school), CESTA VON provides each Omama with training and mentorship to build (i) their skills in the standardized implementation of the intervention, (ii) trust-based relationships with families and (iii) organisational skills in managing their schedules according to each family.

The training of Omamas follows a three-step approach:

1. **Central training workshops**: Each Omama attends a five-day long centralised training workshop together with her peers. During this workshop, Omamas are provided information about the importance of promoting early child development among Roma children, and its potential long term impact on the school, educational and social productivity outcomes of these children. They are then trained by a group of supervisors in each age-appropriate activity using techniques of direct instruction, demonstration and role play. Each Omama is provided a copy of the intervention manual (see figure S3a and S3b below) and mobile-phone based application, which lists details of stimulation activities and the required kit for these for each appropriate age alongside helpful images and videos which serve as a guide to Omamas in the field. During the workshops, each Omama is assigned a mentor – an experienced Omama and/or a trained pedagogist who works alongside each Omama to build her skills in the intervention and provide her with ongoing support.
2. **Field based training:** Each mentor accompanies each Omama over a period of one month to visit families and deliver the intervention. This is led by the Omama with the mentor providing guidance, feedback and support as well as de-briefing Omamas after each session. Mentors focus on building confidence in Omamas about their skills, keeping them motivated and helping them organised more challenging tasks. At times, mentors also assist Omamas will overcoming work-related or personal issues.
3. **Slovak courses:** Most Omamas receive Slovak language courses through CESTA VON’s affiliated AMAL program (<https://cestavon.sk/en/amal/>) where they enhance their spoken and written linguistic skills in Slovak.

CESTA VON has developed a mobile-phone based application (the Omama app) for the Omama program. Omamas are trained to use this application during Phase I. The Omama app facilitates the easy organisation of their working time, and intervention schedules, along with providing Omamas a detailed age-appropriate activity schedule for each child (together with helpful videos and images on the administration of these activities). The application also provides Omamas with a list of activities to share with the family to enable them to continue to provide these to their children during the week. Additionally, the Omama app allows continuous monitoring of children’s development and records any parental or Omama’s concerns regarding neurodevelopmental risks enabling Omamas to rapidly escalate these concerns their mentors for further actions.

**Ongoing Mentorship and Supervision:**

1. **Weekly mentorship sessions:** Following their training, mentors meet with Omamas weekly and provide them with education, intensive mentoring and supervision.
2. **Fortnightly supervision:** Each Omama is also assigned a supervisor who observes the administration of the intervention by each Omama twice monthly. They then provide structured oral and written feedback aimed at the Omama’s professional growth and focussing on the adherence to the program’s methodology.

**Kit:** Most activities utilise simple household items like buckets, boxes, spoons, coloured cloths and basic food items. Additionally Omamas also have access to a few educational toys (such as contrast cards, crayons and a play tunnel) and children’s books.

**Assessment of Adherence to the Intervention Protocol:**

Omamas’ adherence to protocol is recorded on the Omama app. Protocol adherence is reviewed at the weekly meeting Omamas have with their mentors. In addition, each Omama undergoes fortnightly supervision where dedicated supervisors observe the administration of the intervention by each Omama twice monthly and provide structured oral and written feedback.

**Motivation of Omamas:**

Omamas are employed either full or part-time to deliver the intervention by the NGO CESTA VON and they receive a salary for their efforts. They remain motivated to continue these efforts for a number of reasons including obtaining a stable source of income and employment, the opportunity to work with and for their local communities, not having to travel long distances from their communities for employment and their sense of purpose in being directly responsible for enabling a positive change in their communities. Omamas attend initial centralised training sessions, following which they are assigned a mentor who provides them with education, intensive mentoring and supervision on a weekly basis ensuring they remain motivated and helping them troubleshoot problems as they arise.


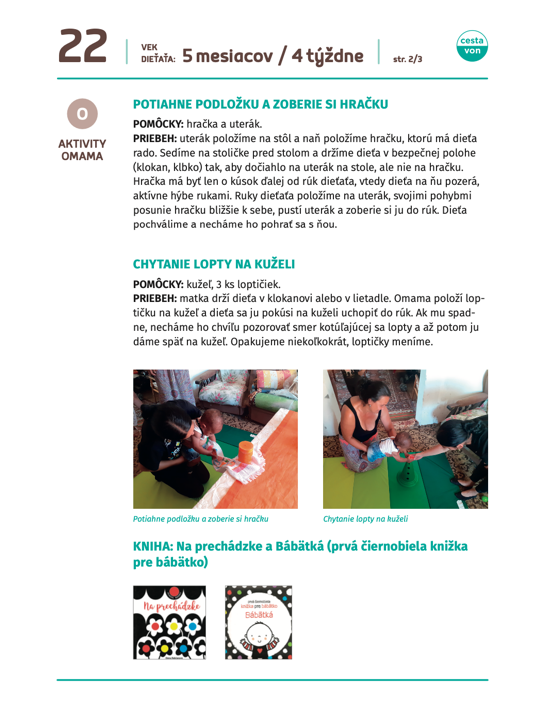

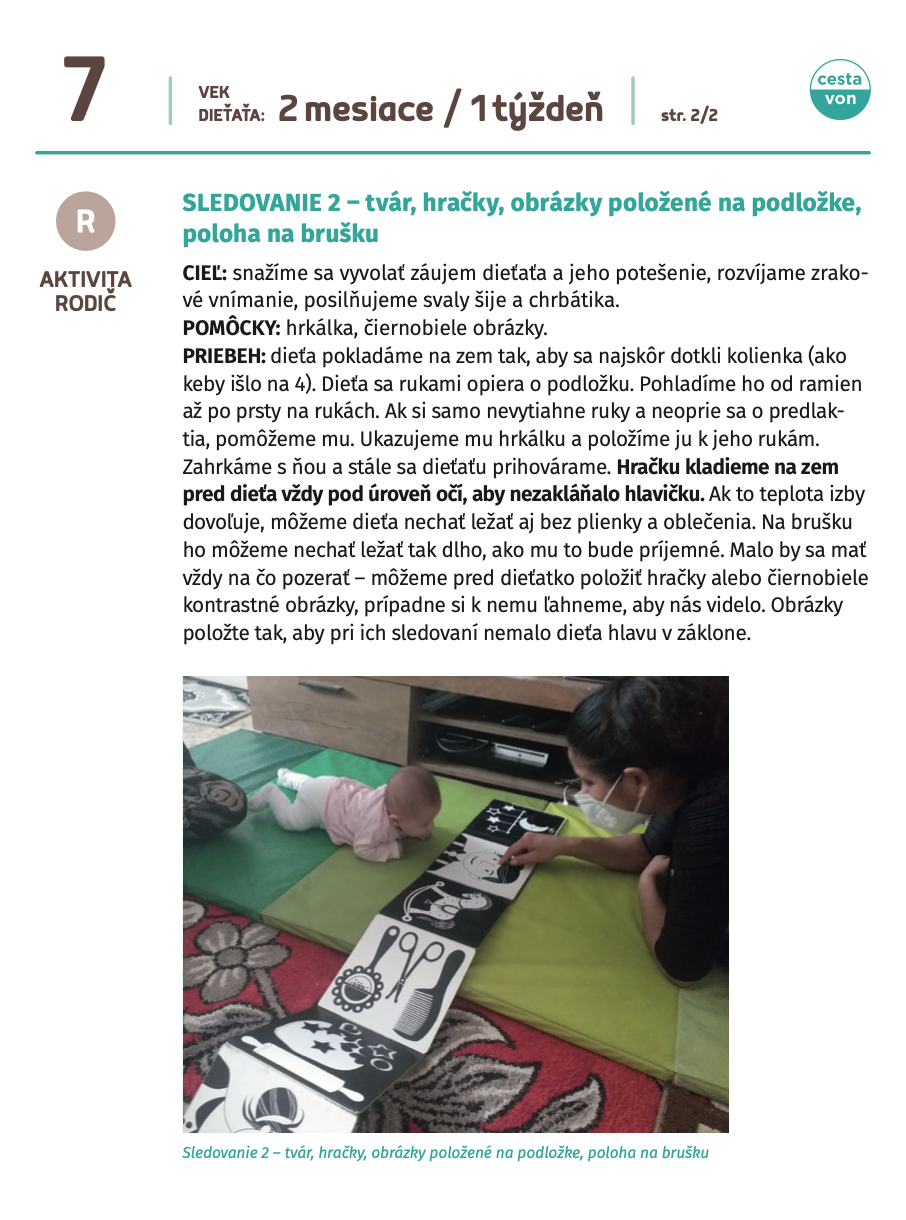


Figures S3b

Figures S3a

**Figures S3a and S3b** Examples of instructions for the intervention from the Omama Project’s intervention manual for children aged 2 months (S3a) and 5 months (S3b)

**Supplementary Information Figure S4** Comparison of growth indices between groups according to the WHO International Child Growth Standards

**RI**

**RC**

**NRC**


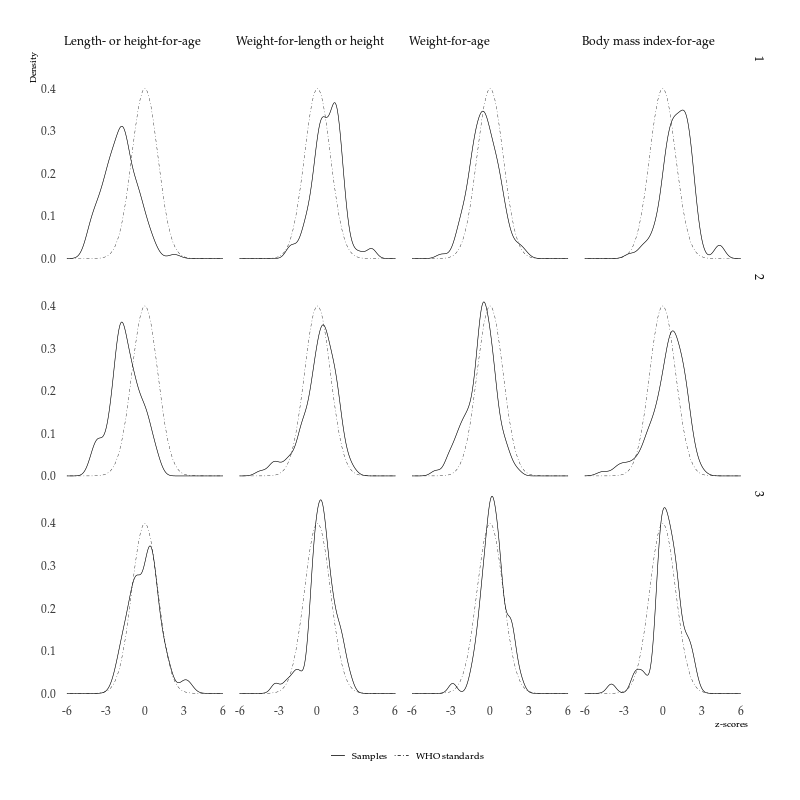


NRC: Non-Roma Control, RI: Roma Intervention, RC: Roma Control.

Results generated using the The WHO Anthro Software: https://www.who.int/tools/child-growth-standards/sof

**Supplementary Information Table S5** Family and home environment characteristics of children in the Omama project cross-sectional ECD study at age 2 years

| **Family and home environment characteristics** | | **Pooled sample** | | | **Roma control (RC) group (n=99)** | | | **Roma intervention (RI) group (n=98)** | | **Non-Roma control (NRC) group (n=54)** | | **Test statistic, p value** | | | |
| --- | --- | --- | --- | --- | --- | --- | --- | --- | --- | --- | --- | --- | --- | --- | --- |
|  |  |  |  |  |  |  |  |  |  |  |  | **RC vs RI comparison** | | **RC, RI v NRC comparison** | |
| **Family Indicators (Mean (SD) or number (%))** | | | | | | | | | | | | | | | |
| Maternal age at birth (in years, Mean (SD)) | | 25.8 (6.9) | | | 23.8 (3.9) | | | 24.1 (7.8) | | 35.0 (1.4) | | t=-0.9, p=0.18 | | F=49.9, p<0.001* | |
| Paternal age at birth (in years, Mean (SD)) | | 28.1 (7.3) | | | 26.2 (5.7) | | | 25.9 (7.5) | | 40.5 (3.5) | | t=-0.8, p=0.20 | | F=47.7, p<0.001* | |
| Mother employed outside house (n (%)) | | 57 (22.7%) | | | 8 (8.2%) | | | 3 (3.1%) | | 46 (85.2%) | | X^2^=2.4, p=0.13 | | X^2^=152.3, p<0.001* | |
| Father employed (n (%)) | | 125 (49.9%) | | | 37 (37.8%) | | | 35 (35.7%) | | 53 (98.1%) | | X^2^=0.1, p=0.77 | | X^2^=63.9, p<0.001* | |
| Permanent paternal employment (n (%)) | | 71 (28.3%) | | | 12 (12.2%) | | | 8 (8.2%) | | 51 (94.4%) | | X^2^=1.3, p=0.87 | | X^2^=149.1, p<0.001* | |
| Number of siblings (n (%)) | | 2.13 (2.22) | | | 2.3 (1.9) | | | 2.4 (2.5) | | 0.5 (0.7) | | t=0.8, p=0.23 | | F=12.9, p<0.001* | |
| Number of siblings aged under 2 years (n (%)) | | 0.19 (0.4) | | | 0.2 (0.4) | | | 0.2 (0.4) | | 0.0 (0.0) | | t=-0.2, p=0.43 | | F=49.9, p<0.001* | |
| Maternal smoking (n (%)) | | 106 (42.3%) | | | 52 (53.6%) | | | 52 (53.1%) | | 2 (3.7%) | | X^2^=0.01, p=0.94 | | X^2^=42.6, p<0.001* | |
| Maternal alcohol consumption (n (%)) | | 43 (17.2%) | | | 15 (15.5%) | | | 17 (17.3%) | | 11 (20.4%) | | X^2^=0.1, p=0.72 | | X^2^=0.6, p=0.75 | |
| **Housing Indicators (Mean (SD) or number (%))** | | | | | | | | | | | | | | | |
| Number of rooms in house (Mean (SD)) | | 2.6 (1.5) | | | 2.3 (1.6) | | | 2.4 (0.7) | | 2.5 (0.7) | | t=-0.3, p=0.38 | | F=0.8, p=0.44 | |
| Number of residents in house (incl. children) (Mean (SD)) | | 6.2 (3.0) | | | 6.2 (2.1) | | | 7.3 (4.2) | | 3.5 (0.7) | | t=-0.2, p=0.41 | | F=49.9, p<0.001* | |
| Room:people ratio (Mean (SD)) | | 3.8 (2.9) | | | 3.7 (2.9) | | | 3.2 (2.2) | | 1.5 (0.7) | | t=0.8, p=0.21 | | F=38.9, p<0.001* | |
| Number of bathrooms/toilets in house (Mean (SD)) | | 0.6 (0.5) | | | 0.3 (0.5) | | | 0.3 (0.5) | | 1.0 (0.0) | | t=-0.9, p=0.17 | | F=23.1, p<0.001* | |
| Cooking occurs in a room where the child sleeps (n (%)) | | 89 (35.5%) | | | 46 (47.4%) | | | 42 (51.9%) | | 1 (1.9%) | | X^2^=0.4, p=0.56 | | X^2^=40.0, p<0.001* | |
| Type of cooking fuel (n (%)):  Wood  Gas  Electric  Oil | | 152 (60.6%)  31 (12.9%)  5 (2.0%)  1 (0.4%) | | | 70 (94.6%)  3 (4.1%)  0 (0%)  1 (1.4%) | | | 75 (97.4%)  1 (1.3%)  1 (1.3%)  0 (0%) | | 7 (18.4%)  27(71.1%)  4 (10.5%)  0 (0%) | | X^2^=3.1, p=0.37 | | X^2^=122.5, p<0.001* | |
|  | *p<0.05 | |  |  | |  |  | |  | |  | |  | |  |

**Supplementary Information Table S6** Three-group comparisons of neurodevelopmental scores of children in the Omama project cross-sectional ECD study at age 2 years

| **Multiple Comparisons** | | | | | | | |
| --- | --- | --- | --- | --- | --- | --- | --- |
| Bonferroni Correction | | | | | | | |
| INTER-NDA standardized domain score | Group comparisons | | Mean Difference (I-J) | Std. Error | Sig. | 95% Confidence Interval | |
|  |  |  |  |  |  | Lower Bound | Upper Bound |
| Cognition | RC | RI | -13.53^*^ | 2.45 | <.001 | -19.43 | -7.63 |
|  |  | NRC | -25.75^*^ | 2.91 | <.001 | -32.76 | -18.75 |
|  | RI | RC | 13.53^*^ | 2.45 | <.001 | 7.63 | 19.43 |
|  |  | NRC | -12.22^*^ | 2.91 | <.001 | -19.24 | -5.21 |
|  | NRC | RC | 25.75^*^ | 2.91 | <.001 | 18.75 | 32.76 |
|  |  | RI | 12.22^*^ | 2.91 | <.001 | 5.21 | 19.24 |
| Language | RC | RI | -15.80^*^ | 3.04 | <.001 | -23.12 | -8.48 |
|  |  | NRC | -31.55^*^ | 3.61 | <.001 | -40.25 | -22.86 |
|  | RI | RC | 15.80^*^ | 3.04 | <.001 | 8.48 | 23.12 |
|  |  | NRC | -15.74^*^ | 3.61 | <.001 | -24.46 | -7.04 |
|  | NRC | RC | 31.55^*^ | 3.61 | <.001 | 22.86 | 40.25 |
|  |  | RI | 15.74^*^ | 3.61 | <.001 | 7.04 | 24.46 |
| Fine Motor | RC | RI | -8.63^*^ | 2.14 | <.001 | -13.80 | -3.47 |
|  |  | NRC | -14.84^*^ | 2.54 | <.001 | -20.97 | -8.71 |
|  | RI | RC | 8.63^*^ | 2.14 | <.001 | 3.47 | 13.80 |
|  |  | NRC | -6.20^*^ | 2.55 | 0.05 | -12.35 | -0.06 |
|  | NRC | RC | 14.84^*^ | 2.54 | <.001 | 8.71 | 20.97 |
|  |  | RI | 6.20^*^ | 2.55 | 0.05 | 0.06 | 12.35 |
| Gross Motor | RC | RI | -10.27^*^ | 2.39 | <.001 | -16.03 | -4.51 |
|  |  | NRC | -13.40^*^ | 2.84 | <.001 | -20.24 | -6.56 |
|  | RI | RC | 10.27^*^ | 2.39 | <.001 | 4.51 | 16.04 |
|  |  | NRC | -3.13 | 2.84 | 0.82 | -9.98 | 3.73 |
|  | NRC | RC | 13.40^*^ | 2.84 | <.001 | 6.56 | 20.24 |
|  |  | RI | 3.13 | 2.84 | 0.82 | -3.73 | 9.98 |
| Positive Behaviour | RC | RI | -5.07 | 3.59 | 0.48 | -13.72 | 3.57 |
|  |  | NRC | -10.43^*^ | 4.26 | 0.05 | -20.70 | -0.17 |
|  | RI | RC | 5.07 | 3.59 | 0.48 | -3.57 | 13.72 |
|  |  | NRC | -5.37 | 4.27 | 0.63 | -15.65 | 4.92 |
|  | NRC | RC | 10.43^*^ | 4.26 | 0.05 | 0.17 | 20.70 |
|  |  | RI | 5.37 | 4.27 | 0.63 | -4.92 | 15.65 |
| Negative Behaviour | RC | RI | 2.82 | 3.67 | 1.00 | -6.04 | 11.67 |
|  |  | NRC | 10.10 | 4.36 | 0.06 | -0.41 | 20.62 |
|  | RI | RC | -2.82 | 3.67 | 1.00 | -11.67 | 6.04 |
|  |  | NRC | 7.28 | 4.37 | 0.29 | -3.25 | 17.82 |
|  | NRC | RC | -10.10 | 4.36 | 0.06 | -20.62 | 0.41 |
|  |  | RI | -7.28 | 4.37 | 0.29 | -17.82 | 3.25 |
| *. The mean difference is significant at the 0.05 level. | | | | | | | |

**Supplementary Information Table S7** Unadjusted comparisons**:** Developmental delay rates by domain at age 2 years

| **INTER-NDA domains** | **Rates of delay**  **n(%)** | **Pooled sample** | **Roma control (RC) group (n=99)** | **Roma interve-ntion (RI) group (n=98)** | **Non-Roma control (NRC) group (n=54)** | **Test statistic, p value** | | |
| --- | --- | --- | --- | --- | --- | --- | --- | --- |
|  |  |  |  |  |  | **RC vs RI comparison** | **NRC vs RI comparison** | **RC, RI v NRC comparison** |
| Cognition^1^ | No delay | 228 (91.2%) | 79 (79.8%) | 95 (97.9%) | 54 (100.0%) | X^2^=16.2 p<0.001* | X^2^=1.1, p=0.29 | X^2^=26.7, p<0.001* |
|  | Any delay | 22 (8.8%) | 20 (20.2%) | 2 (2.1%) | 0 (0.0%) |  |  |  |
| Language^1^ | No delay | 236 (94.0%) | 85 (85.9%) | 97 (99.0%) | 54 (100.0%) | X^2^=12.1, p<0.001* | X^2^=0.6, p=0.46 | X^2^=19.5, p<0.001* |
|  | Any delay | 15 (6.0%) | 14 (14.1%) | 1 (1.0%) | 0 (0.0%) |  |  |  |
| Fine motor^1^ | No delay | 249 (99.2%) | 97 (98.0%) | 98 (100.0%) | 54 (100.0%) | X^2^=2.0, p=0.16 | ^Not computed | X^2^=3.1, p=0.13 |
|  | Any delay | 2 (0.8%) | 2 (2.0%) | 0 (0.0%) | 0 (0.0%) |  |  |  |
| Gross motor^1^ | No delay | 239 (95.2%) | 89 (89.9%) | 96 (98.0%) | 54 (100.0%) | X^2^=5.6, p=0.02* | X^2^=1.1, p=0.29 | X^2^=10.5, p=0.005* |
|  | Any delay | 12 (4.8%) | 10 (10.1%) | 2 (2.0%) | 0 (0.0%) |  |  |  |
| Positive behaviour ^1^ | No problems | 213 (84.9%) | 76 (76.8%) | 85 (86.7%) | 52 (96.3%) | X^2^=3.3, p=0.07 | X^2^=3.6, p=0.06 | X^2^=10.8, p=0.004* |
|  | Any problems | 38 (15.1%) | 23 (23.2%) | 13 (13.3%) | 2 (3.7%) |  |  |  |
| Negative behaviour^2^ | No problems | 191 (76.1%) | 71 (71.7%) | 73 (74.5%) | 47 (87.0%) | X^2^=0.2, p=0.66 | X^2^=3.3, p=0.07 | X^2^=4.7, p=0.09 |
|  | Any problems | 60 (23.9%) | 28 (28.3%) | 25 (25.5%) | 7 (13.0%) |  |  |  |

^1^The threshold for any delay in these domains is defined as standardized INTER-NDA domain scores $\leq$10^th^ centile on the INTER-NDA standards.

^2^For negative behaviour, ‘any’ problems are defined as standardized INTER-NDA domain scores $\geq$90^th^ centile on the INTER-NDA standards.

*p<0.05

^Chi-Square statistic not computed as percentages are identical in cells being tested

**Supplementary Information Table S8** Unadjusted comparisons**:** Developmental delay rates by domain and severity at age 2 years

| **INTER-NDA domains** | **Rates of delay**  **n(%)** | **Pooled sample** | **Roma control (RC) group (n=99)** | **Roma intervention (RI) group (n=98)** | **Non-Roma control (NRC) group (n=54)** | **Test statistic, p value** | | |
| --- | --- | --- | --- | --- | --- | --- | --- | --- |
|  |  |  |  |  |  | **RC vs RI comparison** | **NRC vs RI comparison** | **RC, RI v NRC comparison** |
| Cognition^1^ | No delay | 228 (91.2%) | 79 (79.8%) | 95 (97.9%) | 54 (100.0%) | X^2^=16.4, p<0.001* | X^2^=1.1, p=0.29 | X^2^=27.0, p<0.001* |
|  | Mild to moderate delay | 5 (2.0%) | 5 (5.1%) | 0 (0.0%) | 0 (0.0%) |  |  |  |
|  | Severe delay | 17 (6.8%) | 15 (15.2%) | 2 (2.1%) | 0 (0.0%) |  |  |  |
| Language^1^ | No delay | 236 (94.0%) | 85 (85.9%) | 97 (99.0%) | 54 (100.0%) | X^2^=12.2, p=0.002* | X^2^=0.6, p=0.46 | X^2^=19.6, p<0.001* |
|  | Mild to moderate delay | 10 (4.0%) | 9 (9.1%) | 1 (1.0%) | 0 (0.0%) |  |  |  |
|  | Severe delay | 5 (2.0%) | 5 (5.1%) | 0 (0.0%) | 0 (0.0%) |  |  |  |
| Fine motor^1^ | No delay | 249 (99.2%) | 97 (98.0%) | 98 (100.0%) | 54 (100.0%) | X^2^=2.0, p=0.16 | ^Not computed | X^2^=3.1, p=0.21 |
|  | Mild to moderate delay | 0 (0.0%) | 0 (0.0%) | 0 (0.0%) | 0 (0.0%) |  |  |  |
|  | Severe delay | 2 (0.8%) | 2 (2.0%) | 0 (0.0%) | 0 (0.0%) |  |  |  |
| Gross motor^1^ | No delay | 239 (95.2%) | 89 (89.9%) | 96 (98.0%) | 54 (100.0%) | X^2^=6.6, p=0.04* | X^2^=1.1, p=0.29 | X^2^=11.8, p=0.02* |
|  | Mild to moderate delay | 7 (2.8%) | 5 (5.1%) | 2 (2.0%) | 0 (0.0%) |  |  |  |
|  | Severe delay | 5 (2.0%) | 5 (5.1%) | 0 (0.0%) | 0 (0.0%) |  |  |  |
| Positive  behaviour^1^ | No problems | 213 (84.9%) | 76 (76.8%) | 85 (86.7%) | 52 (96.3%) | X^2^=3.95, p=0.14 | X^2^=4.35, p=0.11 | X^2^=12.51, p=0.01* |
|  | Mild to moderate problems | 18 (7.2%) | 9 (9.1%) | 7 (7.1%) | 2 (3.7%) |  |  |  |
|  | Severe problems | 20 (8.0%) | 14 (14.1%) | 6 (6.1%) | 0 (0.0%) |  |  |  |
| Negative behaviour^2^ | No problems | 191 (76.1%) | 71 (71.7%) | 73 (74.5%) | 47 (87.0%) | X^2^=1.3, p=0.52 | X^2^=3.7, p=0.15 | X^2^=6.8, p=0.15 |
|  | Mild to moderate problems | 53 (21.1%) | 23 (23.2%) | 23 (23.5%) | 7 (13.0%) |  |  |  |
|  | Severe problems | 7 (2.8%) | 5 (5.1%) | 2 (2.0%) | 0 (0.0%) |  |  |  |

^1^The threshold for severe and mild-to-moderate delay in these domains is defined as standardized INTER-NDA domain scores $\leq$3^rd^ and 3^rd^-10^th^ centiles, respectively, on the INTER-NDA standards.

^2^For negative behaviour, severe and mild-to-moderate problems are defined as standardized INTER-NDA domain scores $\geq$97^th^ and 90^th^-97^th^ centiles, respectively, on the INTER-NDA standards.

*p<0.05

^Chi-Square statistic not computed as percentages are identical in cells being tested
